# *Chlamydia* and Its Many Ways of Escaping the Host Immune System

**DOI:** 10.1155/2019/8604958

**Published:** 2019-08-06

**Authors:** Won Fen Wong, James P. Chambers, Rishein Gupta, Bernard P. Arulanandam

**Affiliations:** ^1^South Texas Center for Emerging Infectious Diseases and Center of Excellence in Infection Genomics, University of Texas at San Antonio, One UTSA Circle, San Antonio, TX 78249, USA; ^2^Department of Medical Microbiology, Faculty of Medicine, University of Malaya, Kuala Lumpur 50603, Malaysia

## Abstract

The increasing number of new cases of* Chlamydia* infection worldwide may be attributed to the pathogen's ability to evade various host immune responses. Summarized here are means of evasion utilized by* Chlamydia* enabling survival in a hostile host environment. The pathogen's persistence involves a myriad of molecular interactions manifested in a variety of ways,* e.g*., formation of membranous intracytoplasmic inclusions and cytokine-induced amino acid synthesis, paralysis of phagocytic neutrophils, evasion of phagocytosis, inhibition of host cell apoptosis, suppression of antigen presentation, and induced expression of a check point inhibitor of programmed host cell death. Future studies could focus on the targeting of these molecules associated with immune evasion, thus limiting the spread and tissue damage caused by this pathogen.

## 1. Introduction

Chlamydiae are obligate, intracellular bacteria that target epithelial cells at different mucosal sites and give rise to a wide range of clinical presentations [1]. The most common species of the genus* Chlamydia* that colonizes the human host causing disease is* Chlamydia trachomatis *[2]. Depending on the bacterial outer membrane genotype,* C. trachomatis* isolated from patients is categorized into several different serovars that have been shown to target distinct tissues giving rise to different clinical presentations. Serovar types A, B, and C infect conjunctiva epithelial cells, and if left untreated can cause Trachomatous Trichiasis which can lead to irreversible corneal scarring and blindness [3]. Genital chlamydial infection is caused by* C. trachomatis* serovars D through K. Genital* C. trachomatis* is ranked as the most frequent sexually transmitted bacterial agent worldwide. The ability of* C. trachomatis *serovars D through K to ascend the upper genital tract leads to tubal inflammation, ectopic pregnancy, spontaneous abortion, and infertility in females [4, 5]. Conversely, infection with* C. trachomatis* serovars L1, 2, and 3 is able to spread to nearby lymph node tissue giving rise to Lymphogranuloma venereum (LGV) [6].* C. pneumoniae* is another member species of the genus* Chlamydia* and is associated with pulmonary infection [7].

Upon infection,* Chlamydia *sp. can persist for long periods resulting in unevenly distributed, chronic inflammation of infected tissues, and long-term sequela [8]. The asymptomatic nature of chlamydial infection often leads to delayed diagnosis, and lack of proper antibiotic therapy results in to severe tissue damage [9]. This is a major contributing factor for increased prevalence and transmission of* Chlamydia *sp. infection in recent years. This situation is worrisome as there is no effective prophylactic vaccine available necessitating further investigation for better understanding of the host response to this bacterium. This review provides insight into the molecular means utilized by chlamydial species to the evade immune response.

## 2. The* Chlamydia* Life Cycle and ‘Persistence' in Host Cells

A characteristic of many pathogens is that of ‘persistence',* i.e*., the continued presence of the pathogen under stressful conditions such as limitation of required nutrient(s) and/or presence of antimicrobial/reagents or immune cells [8]. During the 'persistence' period, the pathogen remains viable but discontinues cell development and reproduction. In this stage, the pathogen is noninfectious and as such undetected by the host immune system. This stage will continue until which time a more favorable environment is re-established. Thus, in 'hide and seek' fashion, the pathogen reemerges once the immune system has been evaded and/or deceived at the infection site.

*Chlamydia *sp. has a unique biphasic life cycle; wherein, it alternates to and from ‘Elementary Body' and ‘Reticulate Body' forms,* i.e*., EB and RB, respectively [2]. The EB form is infectious and is metabolically inactive with a rigid outer membrane facilitating binding to and entry into the host cell. Following host cell entry, internalized EB fuses to form an intracytoplasmic inclusion which gives rise to the RB form. Although the RB form is noninfectious, it is metabolically active, and within eight hours* after *infection begins to multiply followed by release within 24 hours infecting neighboring cells [10–12]. The EB form differs from that of the RB in size,* i.e*., the EB is much smaller (0.2 *μ*m) compared to RB (0.8 *μ*m) [13]. The EB is often present in semen and/or female genital tract epithelial cell secretions, and thus is transmitted to partners during sexual intercourse [14]. Although the EB form first binds to the epithelial cell surface heparin sulfate proteoglycan [15], it readily interacts with other surface molecules such as the mannose receptor [16] or glycosylation-dependent galectin–receptor [15] to trigger and facilitate internalization.

*Chlamydia *sp. become persistent,* i.e*., enter the ‘persistence' stage between EB and RB stages enabling the bacterium to survive during unfavorable conditions facilitating its long-term survival in the host,* e.g*., cellular stress associated with immunological host response eliciting proinflammatory cytokines, antibodies, and antimicrobial substances [8]. Once the required nutrient,* e.g.,* amino acid, or immunological host response mediator molecule(s) return to normal prestress levels, the 'persistence' phase is no longer needed.

**Figure 1:**
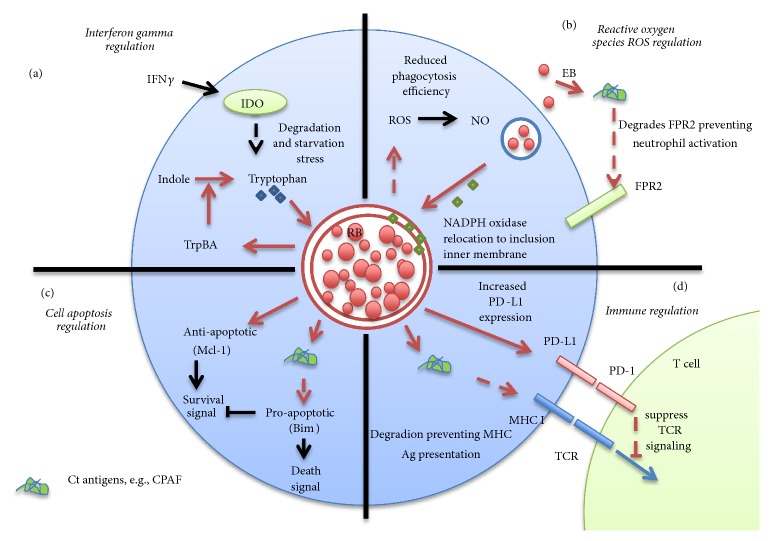
Immune evasion tactics utilized by* Chlamydia *sp. (a) Release of interferon *γ* (IFN*γ*) from immune cells induces expression of* Indoleamine 2,3-dioxgenase* (IDO) which degrades tryptophan, an essential amino acid required for* Chlamydia *sp. replication. IDO-mediated tryptophan depletion gives rise to bacterial stress,* i.e*., starvation. Under such conditions,* Chlamydia *sp. can produce* tryptophan synthase* (TrpBA) that converts indole to tryptophan. To avoid a ‘continuous' stress situation,* Chlamydia *sp. enters a ‘persistence' stage until which time the supply of tryptophan is restored. (b)* Chlamydia *sp. suppresses production of reactive oxygen species (ROS) and nitric oxide (NO) reducing the efficiency of phagocyte bacterial killing in phagolysosome.* NADPH oxidase* which is typically located on the phagolysomal membrane assists in production of bactericidal* Myeloperoxidase* (MPO) and hypochlorous acid (HOCl^−^). In* Chlamydia*-infected cells, the subunit of* NADPH oxidase* is relocated to the inner membrane of the inclusion rather than the phagolysosome. As a consequence,* Chlamydia *sp. are able to survive in the phagocyte. In the neutrophil, production of chlamydial antigens such as* Chlamydial*-protease-like activity factor (CPAF) causes degradation of neutrophil surface* formal peptide receptor* inhibiting activation of neutrophils impeding neutrophil extracellular trap (NET) activity. (c) Release of CPAF from* Chlamydia *sp. induces expression of antiapoptotic molecule* myeloid leukemia cell differentiation protein* (Mcl-1) promoting degradation of proapoptotic molecules such as* BCL-2-like protein 11* (Bim). Thus,* Chlamydia *sp. block host cell apoptosis leading to a longer period of persistence,* i.e*., replication within host cells. (d) CPAF degrades the major histocompatibility complex (MHC) preventing antigen presentation to T cells. Additionally,* Chlamydia *sp. increases PD-L1 expression in host cells. Binding of PD-L1 to the PD-1 receptor on the T cell surface constitutes a negative signal suppressing T cell receptor (TCR) activation signaling. Broken arrows denote degradation.

For example, IFN-*γ* secreted from immune cells promotes* C. trachomatis* entry into the ‘persistence' stage. IFN-*γ* induces expression of* Indoleamine-2,3-dioxygenase* (IDO) enzyme which degrades and thus depletes tryptophan which is required for* C. trachomatis* growth (Figure 1(a)) [17, 18]. Therefore, the presence of host IDO brings about amino acid deprivation,* i.e*., stress that can lead to death and clearance of the pathogen. In order to avoid this specific stress scenario,* C. trachomatis* enters a ‘persistence' phase that negates the need to consume tryptophan becoming undetectable by immune cells [19]. Conversely, reduced IFN-*γ* production and concomitant increased tryptophan concentration promote* C. trachomatis* reverting to its normal RB-EB life cycle, and may lead to recurrences in patients [20]. Additionally,* C. trachomatis* avoids tryptophan depletion via release of* Tryptophan synthase* (TrpBA) protein [21, 22]. The *α*-subunit of the* Tryptophan synthase* converts indole glycerol 3-phosphate (IGP) to indole; whereas, the *β*-subunit converts indole into tryptophan. In the genital tract, this protein induces tryptophan storage, thus providing a continuous supply of tryptophan required for bacteria metabolism.

Even in the ‘persistence' stage,* C. trachomatis* can cause damage to the host. Although* C. trachomatis* discontinues production of most structural and membrane components, it has been shown to synthesize and release a 60 kDa heat shock protein (Hsp60). The presence of Hsp60 protein is thought to cause trophoblast apoptosis leading to fallopian tube epithelial cell damage, and scar formation [23]. Since chlamydial Hsp60 shares high homology with that of human Hsp60 protein produced by human embryonic cells, the immune response elicited against chlamydial Hsp60 is thought to harm the developing embryo leading to spontaneous abortion. However, clinical data have yet to demonstrate a correlation between Hsp60 antibodies and recurrent abortion [24].

## 3. Chlamydial Infection Paralyses Neutrophil Extracellular Trap Formation

A number of studies have focused on characterization of polymorphic nuclear leukocytes (PMNs) or neutrophils in the pathologies caused by* Chlamydia *sp. given that a rapid influx of neutrophils frequently accompanies infection of either the genital or pulmonary tracts. Bacteria eradication by neutrophils usually include common neutrophil functions,* i.e.,* phagocytosis, release of defensins, and* Neutrophil extracellular trap* (NET) formation. The process of neutrophil extravasation to the mucosal site of infection has been shown to be regulated by* Surface beta-2 integrin* CD18 in addition to cytokines such as IL-8 and IL-17 [25–27]. Lower IL-8 levels result in less efficient neutrophil transendothelial migration through* C. trachomatis*-infected human umbilical vein endothelial cells [26]. Reduced leukocyte influx to the site of chlamydial infection in the genital tract is also detected in IL-17RA deficient mice compared to wild type [27].

Zhang and coworkers have demonstrated recruitment of high numbers of neutrophils to the oviduct following intravaginal inoculation with* C. muridarum* which is associated with more rapid resolving of the hydrosalpinx in different animal models [28]. Conversely, neutrophil depletion using monoclonal antibodies demonstrate approximately 6-fold higher bacterial burden at day 7 following intravaginal bacteria inoculation [25, 29]. Additionally, Lee* et al.* using a similar monoclonal antibody approach to induce neutropenia in mouse demonstrated reduced histopathological parameters, and reduced rates of hydrosalpinx following resolution of the infection [30]. Bai* et al*. propose that neutrophils play a limited role in clearance of bacteria. In* C. trachomatis*-infected C3H mouse lung, severe pathology is observed in contrast to the C57BL/6 mouse model; however, the former displays persistence, and more abundant neutrophil infiltration [31]. Furthermore, using the* C-X-C chemokine receptor 2 motif* (CXCR2) deficient mouse model which is characterized by impaired neutrophil recruitment, no difference in* C. trachomatis* pulmonary infected and uninfected wild type animals is observed [31]. Surprisingly, rather than affording protection, Rodriguez* et al*. demonstrated the presence of GR1+/CD45+ neutrophils at the site of infection, and enhanced bacteria replication in lung epithelial cells with concomitant increased* C. pneumoniae* bacterial burden in infected mice [32]. Failure to recruit neutrophils to the infection site has been suggested to be the primary reason for low bacterial burden, and less pathology in chlamydial infected MycD88-deficient mice [32]. However, the presence of neutrophils in the genital tract can also have negative effects,* i.e*., facilitation of infection by human immunodeficiency virus (HIV) [33].

In addition to infection by* C. trachomatis* and* C. pneumoniae*, neutrophil involvement has been observed to play a role in infection by other species such as* C. psittaci* and* C. caviae*. Greater ability of C57BL/6 mice in eliminating* Chlamydia *sp. is correlated with early neutrophil response as well as cytotoxic T cells [34]. When neutrophils are depleted by administration of RB6-8C5 monoclonal antibody intraperitoneally to* C. psittaci* infected mice, infection-induced abortion is accelerated with infected animals exhibiting a 100-fold higher bacteria burden with widespread necrosis of the uteroplacenta and increased mortality [35]. This could be due to a decrease in the general immune response manifested as a lower number of other leukocytes including macrophages and T cells; however, an altered TH1 response is not observed in the absence of neutrophils, and no clinical changes are observed during secondary infection in neutrophil depleted mice [36]. In ocular* C. caviae* infected guinea pigs with neutrophil depletion, ocular pathology as well as increased serum IgA, IL-5, and TGF-*β* but decreased CCL5 are observed [37].

Recently, Rajeeva* et al*. have suggested a neutrophil evasion strategy utilized by* C. trachomatis* resulting in paralysis of host cell extrusion of NET (which contains chromatin DNA and proteolytic enzymes released by neutrophils during Neptosis cell death to trap and lyse extracellular bacteria) [38]. Cleavage and release of neutrophil* Surface Formyl peptide receptor 2 *(FPR2) by the* Chlamydial-protease-like activity factor* (CPAF) plays a role in this process as a CPAF target affecting oxidative burst interfering with chemical-mediated activation of neutrophils (Figure 1(b)). Increased secretion of specific defensin types,* i.e., Human neutrophil peptides* (HNP1-3), are detected in* C. trachomatis* infected patients with urethritis [39]. However, clinical studies have revealed higher HNP1-3 secretion in the vagina of infected females correlating with a higher risk of endometriosis and bacterial ascension, and pelvic inflammatory disease (PID) pathogenesis [40] supporting a negative rather than protective role for neutrophils.

Neutrophil recruitment to the site of infection is also dependent on the presence of virulence factors,* e*.*g*., the 7.5 kb cryptic plasmid. Infection with a plasmid-bearing* C. trachomatis* strain triggers a more rapid release of soluble factors from oviduct epithelial cells leading to a higher abundance of neutrophils with prolonged survival at the infection site [41], and severe clinical symptoms observed in female patients [22].

## 4. *C. pneumoniae* Hides from Phagocytosis

Oxidative stress via* NADPH oxidase* in human neutrophils or HeLa cells has been shown to be inhibited by* C. trachomatis* infection [42, 43]. The mechanism utilized by* C. trachomatis *involves relocation of* Ras-related C3 botulinum toxin substrate 1* (Rac1), a regulatory subunit of* NADPH oxidase* to the inclusion reducing phagocytosis efficiency (Figure 1(b)) [43]. Fluorescence lifetime imaging data suggest NADPH is relocated to the inner side of the chlamydial inclusion membrane [44] promoting bacterial glycolytic function, affecting in a negative fashion host cell energy generation.

When macrophages are infected with* C. pneumoniae*, reactive oxygen species (ROS) are produced via Ca^2+^ influx, and membrane associated* NADPH oxidase* [45]. Interestingly, levels of ROS in human monocytes in response to* C. pneumoniae* are less intensive than that observed for* C. trachomatis*. Thus,* C. pneumoniae* is able to survive longer than* C. trachomatis* in human monocytes [46].* C. trachomatis* infectivity in monocytes can be restored by treatment with* NADPH oxidase* or* Nitric oxide synthase* inhibitors implying that phagocytic cells utilize ROS and/or nitric oxide (NO) for bacterial eradication [46]. ROS release during chlamydial infection is* Nucleotide-binding oligomerization domain, leucine rich repeat containing X1* (NLRX1) dependent, and is turned on rapidly upon infection, but switched off only a few hours* after* infection [47].* C. trachomatis* selectively stimulates* Myeloperoxidase* release, but not superoxide production by human neutrophils [48].

## 5. *Chlamydia sp.* Inhibits Host Cell Apoptosis

*C. pneumoniae* is able to infect and inhibit host cell apoptosis defense function by lowering Pro*caspase 3* processing with concomitant induction of IL-8; thus, maintaining expression of antiapoptotic* Induced myeloid leukemia cell differentiation protein* (Mcl-1) via activation of PI3K/Akt and ERK1/2 pathways (Figure 1(c)) [49, 50]. This enables the bacterium to reside and hide inside the neutrophil for up to 90 hours compared to 10 hours in noninfected neutrophils [50]. When infected neutrophils undergo apoptosis and are eventually ingested by neighboring macrophages, bacteria are able to replicate and persist longer. Infection of macrophage through apoptotic neutrophils induces* Tumor growth factor-β* (TGF-*β*) secretion compared to TGF-*α* following direct infection of macrophage with bacteria [51] facilitating the hiding of bacteria,* i.e.,* remaining protected when taken up by long-lived macrophages. CPAF contributes to chlamydial antiapoptotic activity by degrading the proapoptotic* BH3-only B-cell lymphoma-2* (BCL-2) subfamily death effector members such as* BCL-2-like protein 11* (BIM),* p53 upregulated modulator of apoptosis* (PUMA), and* BCL-2-associated death promoter* (BAD) [52]. BIM protein has been observed to disappear during chlamydial infection, and this disappearance could be inhibited by proteasome inhibitors [53]. These proapoptotic molecules transmit death signals to mitochondria inhibiting both BCL-2 pro/antiapoptotic molecules which activate proapoptotic* BCL-2-associated X protein* (BAX), and* BCL-2 homologous antagonist killer (BAK)* [54]. Thus, degradation of proapoptotic molecules confers resistance to apoptosis during cellular chlamydial infection.

## 6. *C. trachomatis* Suppresses Class I/II MHC to Avoid Immune Detection by T Cells

Intravaginal inoculation with* C. trachomatis* in the mouse chlamydial model causes recruitment of uterine infiltrate composed of a large number of CD45^+^ mononuclear cells that express surface* Class II major histocompatibility complex* (MHC), and the co-stimulatory CD86 molecule to induce T cell activation [55]. Class II MHC is required for immunity to* C. trachomatis* as evidenced by class II deficiency derived from inactivation of the I-A*β* gene which exhibited a lower concentration of all anti-chlamydia antibody isotypes resulting in failure to resolve the infection compared to wild-type mice after 3 weeks [56]. Likewise, athymic nude or CD4^+^ T cell depleted mice also exhibit a profound delay in infection resolution [55, 56] suggesting that engagement of both antigen presentation and helper T cells is required in resolving* C. trachomatis* infection. Involvement of Class II MHC antigen presentation has also been shown through identification of several chlamydial peptides retrieved from Class II MHC-bound peptides eluted from dendritic cells (DCs) pulsed with live or dead* C. muridarum* elementary bodies (EBs) [57].

Many intracellular pathogens especially viruses have been shown to suppress MHC expression or surface presentation to avoid detection by the adaptive immune system. For example, human cytomegalovirus (HCMV) is able to suppress both Class I and II MHC molecules through the* Unique short-2* (US2) and -11 (US11) proteins which target newly synthesized MHC molecules causing ubiquitination, and relocation to the cytosol for proteasome degradation [58, 59]. In contrast, HIV* negative regulatory factor* (Nef) protein diverts transport of Class I MHC to organelles rather than to the cell surface causing accumulation in cells [60, 61]. Nef also induces immature Class II MHC with invariant chain and accelerates endocytic removal of surface class II MHC molecules [62, 63].

As an intracellular bacterial pathogen, it is likely that* C. trachomatis* avoids immune detection by hiding from or interfering with MHC presentation. During the developmental cycle,* C. trachomatis* remains confined within a protective inclusion-like vacuole avoiding Class I MHC presentation (Figure 1(d)). In 1999, Zhong* et al.* reported* C. trachomatis* to inhibit Class II MHC expression [64]. It has been shown in several cell types (MRC-5 human lung fibroblast, 2C4 mouse B cells, and Hela cervical epithelial cells) that* C. trachomatis* infection blocks interferon-*γ* (IFN-*γ*) inducible class II MHC (HLA-DR) expression [64]. Further investigation has demonstrated Class II MHC expression is inhibited through indirect degradation of the* Upstream Stimulatory Factor-1* (USF-1). USF-1 is a constitutive, ubiquitously expressed transcription factor required for expression of IFN-*γ* induction of* Class II Transactivator* (CIITA) which mediates MHC class II expression. Additional studies have revealed other potential targets of CPAF such as proapoptotic BIM and PUMA [52],* Nuclear Factor-kB* (NF-kB) p65 [65],* MHC-like* Cd1d [66], and* Nectin cell adhesion molecule 1* (NECTIN1) [67].

Subsequent to demonstrating* C. trachomatis*-mediated inhibition of Class II MHC expression, Zhong and colleagues reported in* C. trachomatis* that CPAF inhibits Class I MHC by targeting USF-1 [68]. Both constitutive and IFN-*γ*-induced Class I MHC are inhibited in* Chlamydia* infected cells. CPAF, residing in the host cell cytoplasm during infection is responsible for degrading USF-1 and* Regulatory Factor X5* (RFX5) proteins. As mentioned above, USF-1 regulates class II MHC through CIITA; whereas, RFX5 is a member of the RFX transcription factor complex that is required for binding to the X1 regulatory element upstream of MHC Class I heavy chain, and *β*2-microglobulin (*β*2M) genes [69]. Importantly, CPAF is homologous across species, and recombinant CPAF from* C. pneumoniae* has also been shown to degrade RFX5 impairing Class I MHC expression [70].

*Cluster of differentiation d protein* (CD1d) is a MHC-like molecule expressed by epithelial cells, and binds to and presents glycolipid antigens to natural killer T cells [71, 72]. Interestingly, Kawana* et al.* demonstrated CD1d is downregulated by* C. trachomatis* in human penile urethral epithelial cells [66]. This process also involves CPAF-mediated ubiquitination and degradation of CD1d heavy chain. In chlamydial infected cells, CD1d heavy chains have been shown to relocate to the cytosol and chlamydial inclusion vacuole rather than being transported to the cell surface.

In addition to protease-mediated Class I MHC degradation, Caspar-Bauguil* et al*. reported IL-10 secretion by infected cells could play a role in Class I MHC inhibition [73].* C. pneumoniae* infection of U937 human monocytic cells causes suppression of Class I MHC expression, a reaction that could be reversed by addition of anti-IL-10 neutralizing antibody. Furthermore, addition of recombinant IL-10 alone is able to reduce Class I MHC expression in these cells suppressing bacterial epitope presentation and attenuation of T cell mediated elimination of bacteria.

## 7. Induction of PD-L1 in* Chlamydia* Infected Cells Causes T Cell Exhaustion

Increased expression of* Programmed cell death protein-1* (PD-1) is indicative of T cell exhaustion as evidenced in many types of chronic viral infections [74]. PD-1 binding to its ligands (PD-L1 and PD-L2) on antigen presenting cells suppresses T cell receptor signaling-mediated activation conferring T cell persistence in the ‘exhausted state' which is characterized by unresponsiveness to antigen exposure, loss of cytotoxicity, and cytokine*, i.e.,* IL-2, TNF*α*, and IFN*γ* production [75]. In recent years, antibody and cell immunotherapeutic approaches used to interfere with PD-1 or its ligands have proven to be clinically affective as evidenced by the conferring of the 2018 Nobel Prize in Medicine/Physiology. By targeting PD-1 signaling, T cell exhaustion during chronic infection can be reversed reinvigorating T cell activity for active pathogen clearance. This therapeutic approach initially used in cancer immunotherapy has also been applied in clinical intervention of viral pathogens such as HIV [76]. Most T cell exhaustion studies are conducted using CD8+ cytotoxic T cells, and chronic viral-mediated infection models. However, the focus of a few studies has been on characterization of the involvement of PD-1 signaling in bacterial infection. Given that* Chlamydia* is an obligate intracellular parasite phenotypically analogous to that of the viral life cycle with its long-term host persistence, some studies have begun to elucidate the potential role of PD-1 signaling in the host response to this pathogen (Figure 1(d)).

Although the principal function of cell-mediated immunity is interdiction and eradication of intracellular pathogens, the focus of most chlamydial studies to date is not on CD8^+^ T cell response because CD8+ T cell involvement has been shown to play a minimal role in* C. trachomatis* immunity in genital tract infection in the murine model [77]. Fankhauser* et al.* have attributed poor CD8^+^ T response during genital* C. trachomatis* infection to PD-1 signaling [78]. Having measured the number of immune-dominant antigen* Cysteine-rich membrane protein-* (CrpA-) Class I tetramer specific CD8+ T cells in the genital mucosa, a high number of infiltrating CD8^+^T cells during primary intracervical* C. trachomatis* infection was observed with clearing,* i.e.,* resolving after 4 weeks. However, CD8^+^ T cells were greatly diminished at the genital mucosa upon secondary reinfection after 5 weeks reminiscent of chronic viral pathogen infection [74, 79]. Administration of anti-CD8 depleting antibody shows no difference in the ability to clear bacteria suggesting that memory CD8^+^ T cells have an impaired ability to expand; thus, not contributing to control of* C. trachomatis* during secondary infection. This defective response during secondary infection is attributed to a 10-fold higher expression of PD-L1 in the uterus of infected mice that contributes to impaired bacterial clearance from the host [78]. PD-L1 engagement results in lower IFN-*γ* secretion from CD8+ T cells while inhibition of PD-L1 restores the CD8^+^ response. After primary transcervical infection, PD-L1 deficient mice exhibit lower bacterial load. Thus, deletion or inhibition of the PD1/PD-L1 pathway improves the CD8^+^ T cell response resulting in enhanced bacterial clearance.

In a recent study using a* C. muridarum* mouse lung infection model, Shekhar* et al*. demonstrated PD-L1 expression in two different subsets of pulmonary dendritic cells,* i.e., *CD103^−^CD11b^high^ and CD103^+^CD11b^low^ [80]. CD11b^high^ dendritic cells are associated with effector response and inflammation; whereas, CD103^+^ dendritic cells are linked to T Helper 2 and regulatory T cells [81]. Both populations exhibit equal levels of PD-L1 expression in response to infection. Interestingly, when the PD1/PD-L1 signaling is blocked by anti-PD1 antibodies in an* in vitro* coculture experiment, the ability of dendritic cells to promote IFN-*γ* and IL-17 production and release from CD4^+^ T cells is greatly enhanced [80]. Thus, these observations suggest that antibody treatment to block PD1/PD-L1 signaling could be employed to enhance dendritic cell promotion of the TH1/TH17 response boosting protective immunity to* C. trachomatis* infection.

Conversely, Peng* et al*. demonstrated a contradictory role for PD-1 in* C. muridarum* genital infection [82]. Administration of neutralizing antibodies against PD-L1 and co-inhibitory* T-cell immunoglobulin and mucin-domain containing-3* (TIM3) has no effect on bacteria shedding during early stage infection [82]. However, when mice were harvested at 60 days* after* infection, increased hydrosalpinx scores and severe inflammatory response in the uterine horn and oviduct of the upper genital tract are observed suggesting that PD-1/PD-L1 and Tim3 may negatively regulate pathology attenuation in chronic chlamydial infection. Most likely, the different results observed in genital chlamydial infection following PD-1/PD-L1 signaling interference are attributed to CD8+ T cell involvement in either protection or pathology [83].

As previously indicated, a 7.5 kb cryptic* C. trachomatis* plasmid has been implicated as one of several virulence factors associated with more severe pathology in both human and mouse studies. In a transcriptional profiling analysis, Porcella* et al.* report that plasmid-bearing* C. trachomatis* strains enhance expression of PD-L1 two-fold compared to plasmid-deficient strains in human epithelial cells [84]. In addition to PD-L1, other immune suppression-related molecules,* e.g*.,* NF-κB inhibitor β protein* (NF-*κ*BI*β*), and* Tumor necrosis factor-α inducing protein 3* (TNF*α*IP3) are also expressed at higher levels in epithelial cells infected with plasmid-bearing strains suggesting that one of eight genes encoded by the plasmid may act to switch off specific immune functions underscoring the need to further investigate and better understand the plasmid immune suppression mechanism.

## 8. Conclusion

Despite studies to improve diagnosis, treatment, and vaccine development, the rate of* Chlamydia* infection has steadily increased worldwide in recent years. This review is a summary of various molecules used by* Chlamydia sp.* that facilitate long-term survival and replication in the host cell. It is important to note that the existence of various nonimmune evasion strategies of the bacteria, i.e., the ability of* Chlamydia *sp. to modify the host transcription or proteome profiles [85, 86] is not included in the current review. A better understanding of interactions between* Chlamydia *sp. and host immune cells is essential for development of better and more effective therapeutic strategies for interdiction of chlamydial infection.
